# Diagnostic Performance and Trending Ability of Continuous Non-Invasive Hemoglobin Monitoring During Elective Intracranial Neurosurgery with Invasive Arterial Monitoring: Influence of Anesthetic Technique

**DOI:** 10.3390/diagnostics16050673

**Published:** 2026-02-26

**Authors:** Hatice Eyiol, Oguzhan Arun

**Affiliations:** 1 Department of Anesthesia and Reanimation, Beyhekim Training and Research Hospital, 42100 Konya, Turkey; 2 Department of Anesthesia and Reanimation, Selcuk University, 42100 Konya, Turkey; oguzarun@selcuk.edu.tr

**Keywords:** noninvasive hemoglobin monitoring, SpHb, pulse CO-oximetry, diagnostic agreement, neurosurgery, perfusion index

## Abstract

**Background:** Continuous non-invasive hemoglobin monitoring (SpHb) may provide real-time information during surgery, but its accuracy in neurosurgery remains uncertain. We evaluated the agreement, trending ability, and diagnostic performance of SpHb compared with arterial blood gas hemoglobin during elective intracranial neurosurgery. **Methods:** In this prospective observational study, 60 adults undergoing elective neurosurgery with invasive arterial monitoring were included. SpHb (Masimo Radical-7) was compared with paired arterial hemoglobin values. Agreement was assessed using repeated-measures Bland–Altman analysis and mixed-effects modeling. Trending ability was evaluated using four-quadrant concordance with an exclusion zone of ±0.5 g/dL. Discrimination for severe anemia (Hb < 8 g/dL) was assessed using ROC analysis with patient-level cluster bootstrapping. **Results:** A total of 190 paired measurements were analyzed. Mean bias was +0.23 g/dL, with wide limits of agreement (−3.26 to +3.72 g/dL). Agreement was worse under low-perfusion-index conditions. Trending performance was preserved, with an overall concordance rate of 85.5%. SpHb showed moderate discrimination for severe anemia (AUC 0.78; 95% CI 0.61–0.93), although severe anemia events were infrequent. **Conclusions:** SpHb showed limited reliability for absolute hemoglobin quantification during neurosurgery but provided useful trend information. SpHb should not replace invasive hemoglobin measurements for clinical decision-making.

## 1. Introduction

Hemoglobin remains one of the most frequently requested laboratory tests in hospital practice. Normal hemoglobin concentrations range between 13.5 and 18 g/dL in men and 11.5–16 g/dL in women [1]. Measurement of blood hemoglobin concentration plays a central role in the detection, evaluation, and management of both acute and chronic anemia. According to the World Health Organization (WHO), anemia is defined as a hemoglobin level below 12 g/dL in women and below 13 g/dL in men [2]. Although the primary purpose of hemoglobin measurement is the identification of anemia, it is also widely used to monitor clinical course and to assess the effects of therapeutic interventions.

Complete blood count analysis is considered the gold standard for hemoglobin determination [1]. However, this method has several limitations, including its invasive and painful nature for patients, higher costs, delays associated with laboratory processing, and the fact that it is not part of routine physical examination. A device capable of rapid and accurate hemoglobin measurement would be particularly valuable in settings requiring prompt triage and treatment decisions, such as emergency departments, home healthcare services for chronically ill patients, operating rooms for intraoperative blood loss estimation and transfusion decision-making, and intensive care units. In these settings, devices requiring blood sampling may be impractical or suboptimal. Consequently, noninvasive hemoglobin measurement techniques have been developed. These technologies not only provide rapid hemoglobin estimation but also enable continuous monitoring, allowing detection of real-time changes and assessment of hemoglobin trends over time.

Pulse oximetry, first described by Aoyagi in 1974, rapidly gained widespread acceptance and has become an integral component of routine patient monitoring [3]. Pulse oximeters noninvasively estimate oxygen saturation using light-emitting diodes at red (660 nm) and infrared (950 nm) wavelengths. Oxygen saturation is calculated based on the ratio between the pulsatile and non-pulsatile components of light absorption at these wavelengths. Under normal conditions, this estimation is highly reliable; however, its accuracy may be compromised in situations associated with impaired perfusion, such as hypotension, patient movement, or severe hypoxia [4]. Following encouraging results from studies utilizing multiple wavelengths, the first commercial pulse CO-oximetry device, Rad-57, was introduced into clinical practice in 2005 [5].

In 2008, Masimo Corporation introduced the Masimo Radical-7 Pulse CO-Oximeter, which enables noninvasive continuous measurement of total hemoglobin (SpHb) [4,6]. The Radical-7 device uses a finger probe and analyzes light absorption across more than seven wavelengths to estimate hemoglobin concentration. By comparing the emitted and detected light, the system calculates the amount of light absorbed by tissues, capillaries, arteries, and veins, which is then matched against known absorption spectra.

Although previous studies have demonstrated a good correlation between SpHb and arterial hemoglobin values, SpHb measurements obtained with the Radical-7 system may be affected by inadequate peripheral perfusion related to temperature gradients [7,8,9,10,11,12,13,14,15,16,17].

Several studies have reported reduced measurement reliability at low perfusion index values provided by the device [8,11,14,16].

In addition, inaccurate measurements may occur in the presence of dyshemoglobins, such as carboxyhemoglobin or methemoglobin, or at low hemoglobin concentrations [13,14,16].

Despite constituting only approximately 2% of total body weight, the brain receives 20–25% of cardiac output and consumes nearly 20% of total body oxygen for energy production. The total amount of oxygen delivered to the brain is determined by the product of cerebral blood flow (CBF) and arterial oxygen content. Cerebral blood flow varies according to metabolic activity and cerebral perfusion pressure (CPP). Oxidative metabolism is the primary mechanism of cerebral energy production; therefore, maintenance of adequate cerebral perfusion pressure is critical for optimal brain function and integrity [18].

In critical conditions characterized by increased cerebral oxygen demand but insufficient oxygen delivery due to anemia or hemorrhage, secondary hypoxic brain injury may occur. As with all therapeutic decisions, transfusion strategies should be individualized and based on the patient’s overall clinical condition, including comorbidities and the presence of ongoing bleeding [19].

In this study, the primary aim was to evaluate the diagnostic agreement and accuracy of continuous non-invasive hemoglobin monitoring (SpHb) compared with arterial blood gas-derived hemoglobin measurements during elective neurosurgical procedures. The secondary aims were to assess the ability of SpHb to track directional changes in hemoglobin levels over time (trending ability) and to explore the potential influence of anesthetic technique and peripheral perfusion status on measurement performance. Recent perioperative and diagnostic studies have further highlighted the variability of SpHb accuracy across different clinical settings, emphasizing the need for procedure-specific validation [9,12,13,16,17].

## 2. Materials and Methods

### 2.1. Study Design

This study was conducted in the Neurosurgery operating rooms of the Selçuk University Faculty of Medicine after obtaining approval from the Selçuk University Faculty of Medicine Ethics Committee. A total of 60 patients aged 18 years and older, classified as American Society of Anesthesiologists (ASA) physical status I–III, who were scheduled for elective neurosurgical procedures under general anesthesia were included after obtaining written and verbal informed consent. The study adhered to the ethical principles outlined in the Declaration of Helsinki and was approved by the Selçuk University Faculty of Medicine Ethics Committee (approval number 2019/83, dated 31 January 2019).

No formal a priori sample size calculation was performed, as this was a prospective observational validation study and the primary objective was the estimation of agreement metrics rather than hypothesis testing. The sample size was determined by feasibility during the study period. The final cohort (60 patients contributing 190 paired measurements) was considered adequate to estimate mean bias and limits of agreement using repeated-measures Bland–Altman methods and mixed-effects modeling with acceptable precision.

### 2.2. Demographic Characteristics

Demographic and clinical data, including age, height, weight, body mass index (BMI), sex, comorbid conditions (such as hypertension, diabetes mellitus, coronary artery disease, arrhythmia, asthma, chronic obstructive pulmonary disease, and hyperthyroidism), chronic medications, previous surgical history, and ASA physical status classification, were recorded by the attending anesthesiologist.

### 2.3. Patient Population

Patients aged 18 years and older with an ASA physical status of I–III who routinely underwent invasive arterial blood pressure monitoring and arterial blood gas analysis during elective neurosurgical procedures were eligible for inclusion in the study. The included procedures comprised elective intracranial neurosurgical operations, including tumor resections, aneurysm clipping, decompressive craniectomy, and functional cranial surgeries. Because invasive arterial monitoring and perioperative ABG sampling were part of routine practice for these cases, the study population may represent a higher-risk elective neurosurgical subset. The choice of anesthetic technique was based on clinical considerations (e.g., neurophysiologic monitoring requirements, anesthesiologist preference, and patient-specific factors) rather than random assignment.

### 2.4. Exclusion Criteria

**Figure 1:**
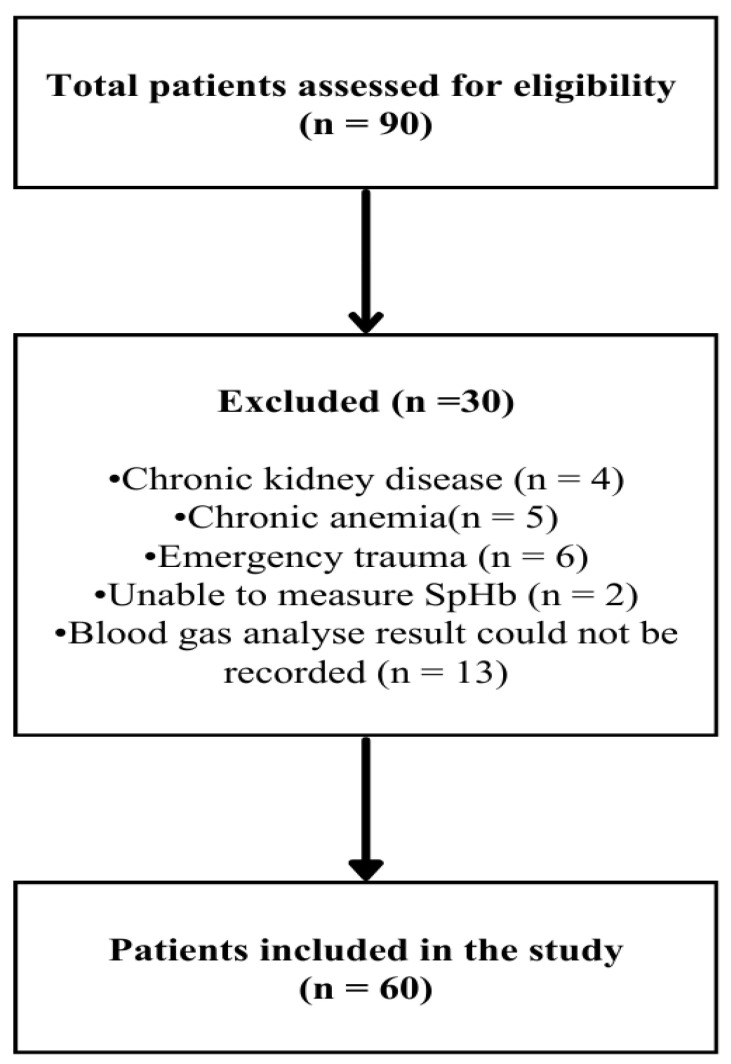
Flowchart of patient selection.

Patients were excluded if they were pregnant; had uncontrolled hypertension (diastolic blood pressure > 110 mmHg and/or systolic blood pressure > 180 mmHg); had significant cardiac pathology (such as electrocardiographic evidence of acute ischemia or decompensated heart failure); had a psychiatric disorder requiring treatment; had active preoperative bleeding, peripheral vascular disease, chronic anemia, or hepatic or renal failure; or were emergency trauma cases. The patient selection process is illustrated in Figure 1 as a flowchart.

### 2.5. Hemoglobin Measurements

Hemoglobin levels were measured using the Radical-7 Pulse CO-Oximeter (Masimo Corp., Irvine, CA, USA) and compared with hemoglobin values obtained from arterial blood gas (ABG) analyses at predefined time points. Measurements were recorded preoperatively, intraoperatively at specified intervals, and at the end of surgery simultaneously with arterial blood gas sampling prior to extubation. All measurements were performed under standardized conditions to minimize external factors affecting SpHb accuracy.

Arterial blood gas sampling was performed at predefined perioperative timepoints (baseline before incision and end of surgery) and additionally when clinically indicated, including suspected bleeding, hemodynamic instability, or at the discretion of the attending anesthesiologist for patient safety. Therefore, paired measurements may over-represent physiologically unstable periods. This approach reflects real-world intraoperative practice but may introduce verification and selection bias, which is addressed through sensitivity analyses.

For analytical purposes, each arterial hemoglobin value was paired with the temporally closest SpHb measurement recorded within a ±2 min window. SpHb values were continuously displayed, and pairing was performed to minimize temporal mismatch between invasive and noninvasive measurements. The distribution of paired measurements spanned the pre-incision, intraoperative, and end-of-procedure periods.

During intraoperative monitoring, sensor repositioning or device recalibration was performed when the perfusion index dropped below 1% or when unstable SpHb readings were observed, in accordance with the manufacturer’s recommendations. These adjustments occurred infrequently and were part of routine clinical practice. Measurements obtained after repositioning or recalibration were not excluded from analysis, as the aim of the study was to evaluate SpHb performance under real-world intraoperative conditions.

Patients with chronic anemia were excluded from the study. Chronic anemia was operationally defined as a previously diagnosed anemia requiring ongoing treatment, a documented hematologic disorder, and/or anemia persisting for more than three months based on available medical records.

In contrast, isolated low preoperative hemoglobin values identified at baseline without documentation of chronic anemia were not considered an exclusion criterion.

The baseline arterial blood gas sample was obtained after induction of anesthesia and arterial cannulation, once hemodynamic conditions were stabilized, but before surgical incision.

### 2.6. Anesthesia Procedures and Data Collection

Upon arrival in the operating room, all patients underwent routine monitoring, including electrocardiography, noninvasive arterial blood pressure, peripheral oxygen saturation (SpO_2_), and axillary body temperature. A minimum fasting period of 6 h was confirmed for all patients. Standard anesthesia induction was performed using intravenous propofol (2–3 mg/kg), rocuronium bromide (0.6 mg/kg), and fentanyl (2 µg/kg). Anesthesia maintenance was achieved using total intravenous anesthesia (TIVA) or inhalational anesthesia with desflurane or sevoflurane in combination with remifentanil. Due to the potential need for blood transfusion, an additional peripheral venous access (16–18 G) was established in the upper extremity.

Following a normal Allen test, radial artery cannulation was performed on either the right or left side to initiate continuous invasive arterial blood pressure monitoring. Heart rate, SpO_2_, systolic, diastolic, and mean arterial pressure, body temperature, fraction of inspired oxygen (FiO_2_), tidal volume, respiratory rate, positive end-expiratory pressure (PEEP), end-tidal carbon dioxide (EtCO_2_), remifentanil/TIVA infusion rate, and minimum alveolar concentration (MAC) values were recorded at 5 min intervals.

Continuous noninvasive hemoglobin (SpHb) measurements were obtained from the third or fourth finger of the hand without radial artery cannulation using a Radical-7^®^ Pulse CO-Oximeter^®^ (Masimo Corp., Irvine, CA, USA), which employs a spectrophotometric method. Sensors were applied according to the manufacturer’s instructions, shielded with opaque covers to prevent optical interference, and their positioning was verified before each measurement. In cases where the perfusion index was <1%, the sensor was repositioned and the device was recalibrated. The initial SpHb measurement was recorded after the device had provided stable readings for at least 15 min.

Prior to surgical incision, a baseline arterial blood gas sample was obtained via the radial artery catheter, and the corresponding SpHb value was recorded simultaneously. During arterial blood gas sampling, SpO_2_, mean arterial pressure, EtCO_2_, and perfusion index values were documented. Arterial blood gas sampling was repeated intraoperatively when clinically indicated, independent of SpHb readings. Hemoglobin and hematocrit values obtained from arterial blood gas analyses were recorded alongside simultaneous SpHb measurements.

In addition, arterial blood gas parameters including SpO_2_, partial pressures of oxygen (PO_2_) and carbon dioxide (PCO_2_), bicarbonate (HCO_3_^−^), base excess, and lactate levels were recorded. Estimated blood loss, amounts of transfused erythrocyte suspension and fresh frozen plasma, duration of surgery, and volumes of administered crystalloid and colloid fluids were documented. At the end of surgery, all data were reviewed, and postoperative laboratory hemoglobin values were added to the study records. The noninvasive hemoglobin sensor was single-use and discarded after completion of the procedure for each patient.

The administration of vasoactive agents was recorded when present and was analyzed in exploratory subgroup analyses, but it was not protocolized or standardized as a primary study variable.

## 3. Statistical Analysis

Statistical analyses were performed using SPSS version 26.0 (IBM Corp., Armonk, NY, USA) and R v4.3.0. Continuous variables were assessed for normality by visual inspection and the Shapiro–Wilk test and are presented as mean ± standard deviation (SD) or median (interquartile range), as appropriate. Categorical variables are expressed as number (percentage). Agreement between SpHb and arterial hemoglobin was assessed using repeated-measures Bland–Altman analysis implemented via linear mixed-effects modeling. Models included a random intercept for each subject to account for within-patient correlation of repeated measurements. Variance components were estimated assuming compound symmetry, and total variance was used to derive 95% limits of agreement with corresponding confidence intervals. To explore the influence of perfusion status, agreement analyses were stratified according to perfusion index (PI), analyzed both as categorical groups and as clinically relevant strata (PI < 1, PI 1–3, and PI > 3). Sensitivity analyses excluding a single extreme outlier were performed to assess the robustness of agreement estimates. Because low perfusion index values occurred in a limited subset of measurements, PI-stratified regression analyses were considered exploratory. Model stability was assessed through sensitivity analyses using alternative PI categorizations and simplified model specifications. Penalized regression approaches were considered; however, given the repeated-measures structure and limited number of low-PI observations, results were primarily interpreted based on effect size direction and confidence intervals rather than statistical significance alone.

Analyses were conducted using available paired observations. No imputation was performed for missing data, as missingness was primarily related to clinical indication for arterial blood gas sampling rather than measurement failure. The number of paired measurements contributing to each analysis is explicitly reported.

Mixed-effects regression models were also used to evaluate determinants of measurement bias, with fixed effects for the perfusion index and time point and random intercepts for subjects. Effect estimates are reported with 95% confidence intervals.

Trending ability was assessed using four-quadrant plot analysis, comparing changes between consecutive paired measurements. An exclusion zone of ±0.5 g/dL was applied a priori to reduce the impact of clinically insignificant changes and random measurement variability. Concordance rates are reported with 95% confidence intervals. Clinical relevance of measurement error was assessed using predefined error thresholds (±0.5, ±1.0, and ±1.5 g/dL) and percent error calculations. To account for within-patient correlation in ROC/AUC analyses, we used patient-level cluster bootstrapping (resampling patients with replacement and retaining all their repeated observations) to obtain robust confidence intervals. As a sensitivity analysis, we repeated ROC/AUC using one clinically relevant observation per patient (the lowest arterial hemoglobin measurement per patient). An absolute difference between SpHb and arterial hemoglobin greater than 1 g/dL was defined as clinically unacceptable disagreement. This threshold was selected based on commonly used criteria in previous perioperative validation studies of non-invasive hemoglobin monitoring, in which differences exceeding 1 g/dL were considered potentially relevant for transfusion-related clinical decision making.

No formal adjustment for multiple testing was applied. Secondary and subgroup analyses, including perfusion index- and anesthetic technique-stratified analyses, were predefined as exploratory, and results were interpreted accordingly with emphasis on effect sizes and confidence intervals rather than statistical significance alone.

All tests were two-sided, and a *p*-value < 0.05 was considered statistically significant.

## 4. Results

### 4.1. Baseline Characteristics

A total of 60 patients were included in the study. The mean age was 60.8 ± 13.9 years, and 30 patients (50.0%) were female. The mean body weight and height were 77.8 ± 12.9 kg and 166.1 ± 9.2 cm, corresponding to an estimated total blood volume of 4587 ± 550 mL. With respect to perioperative risk, 39 patients (65.0%) were classified as ASA physical status I–II and 21 patients (35.0%) as ASA III. The mean number of comorbidities was 1.55 ± 1.22.

The mean duration of surgery was 92.6 ± 28.4 min, and the mean preoperative fasting time was 10.4 ± 2.3 h. Volatile anesthesia was used in the majority of patients (*n* = 55, 91.7%), whereas total intravenous anesthesia was administered in 5 patients (8.3%).

The cohort was exposed to marked perioperative volume shifts. Mean estimated blood loss was 1000.0 ± 707.5 mL, and 26 patients (43.3%) experienced blood loss ≥20% of their estimated total blood volume. The mean total crystalloid administration was 2836.7 ± 1094.9 mL, corresponding to a crystalloid infusion rate of 30.8 ± 13.3 mL/kg/h. Notably, 59 patients (98.3%) received crystalloids at rates exceeding 10 mL/kg/h. Vasopressors were administered in 8 patients (13.3%).

The mean baseline (post-induction, pre-incision) arterial hemoglobin concentration was 11.7 ± 1.8 g/dL, and preoperative anemia (hemoglobin < 12 g/dL) was present in 31 patients (51.7%). This finding reflects perioperative hemoglobin status at the time of surgery and does not indicate chronic anemia, which was excluded by study design. The mean perfusion index (PI) at baseline was 2.83 ± 1.55. Low perfusion index values (PI < 1 at baseline) were observed in 3 patients (5.0%). Repeated intraoperative measurements resulted in additional observations with PI < 1 in a subset of patients; these measurement-level data are reported separately in the PI-stratified analyses (Table 1).

### 4.2. Agreement Between SpHb and Arterial Hemoglobin

Across 190 paired measurements obtained from 60 patients, the mean bias between SpHb and arterial hemoglobin was +0.23 g/dL. The total standard deviation was 1.78 g/dL, yielding limits of agreement ranging from −3.26 to +3.72 g/dL. These findings indicate a small mean bias but wide dispersion of individual measurements (Table 2).

Perfusion index-stratified analyses were based on 37 paired measurements obtained under low-PI conditions (PI < 1) and 152 paired measurements with PI ≥ 1. Given the limited number of low-PI observations, estimates derived from PI-stratified regression models showed wider confidence intervals.

In mixed-effects models, low PI was associated with higher absolute measurement error compared with higher PI, with effect sizes reported alongside 95% confidence intervals (Table 3). These findings indicate a consistent direction of effect, although precision was reduced in the low-PI stratum.

When stratified by perfusion index category, measurements obtained under low-PI conditions demonstrated a higher mean bias and wider limits of agreement compared with those obtained under higher-PI conditions. Specifically, in the low-PI group, the mean bias was 0.32 g/dL with limits of agreement from −3.54 to +4.18 g/dL, whereas in the high-PI group, the mean bias was 0.16 g/dL with limits of agreement from −3.07 to +3.39 g/dL (Table 4).

### 4.3. Mixed-Effects Modeling of Bias

In a linear mixed-effects model accounting for repeated measurements within individuals, neither time nor perfusion index treated as a continuous variable was independently associated with SpHb–arterial hemoglobin bias. However, substantial inter-individual variability was observed, as reflected by a significant random intercept variance.

**Figure 2:**
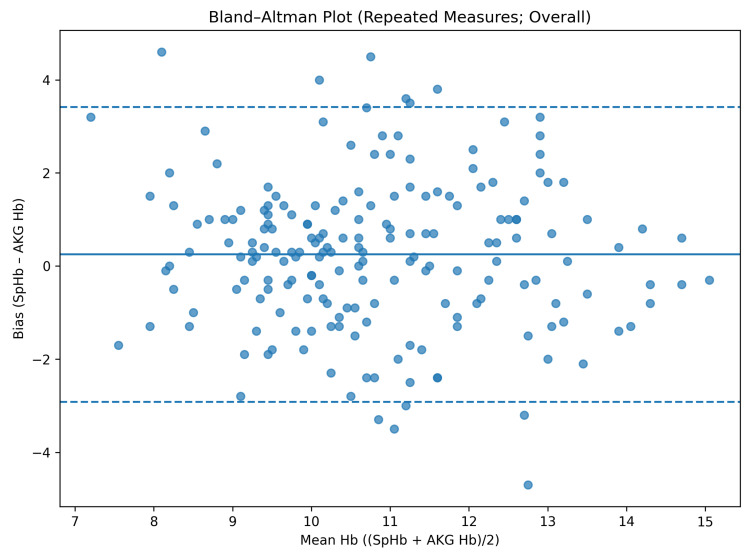
Bland–Altman analysis of agreement between SpHb and arterial hemoglobin measurements (overall). The solid line indicates the mean bias, and the dashed lines indicate the 95% limits of agreement (mean bias ± 1.96 SD).

**Figure 3:**
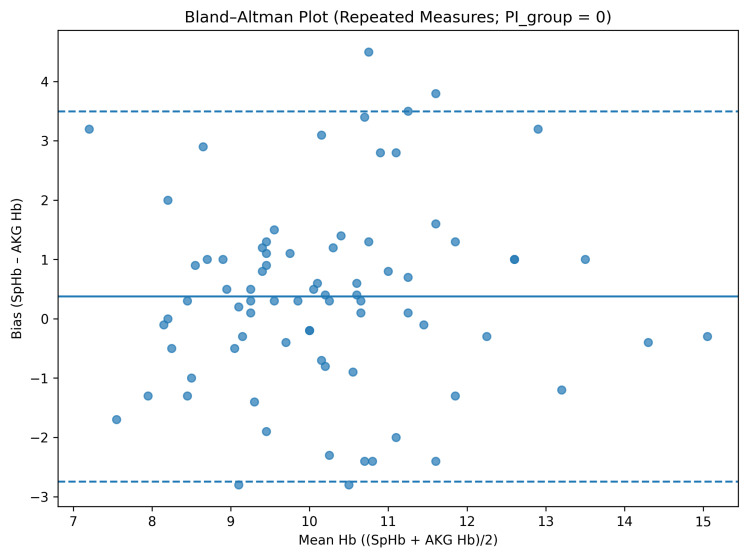
Bland–Altman analysis stratified by perfusion index (PI < 1). The solid line indicates the mean bias, and the dashed lines indicate the 95% limits of agreement (mean bias ± 1.96 SD).

**Figure 4:**
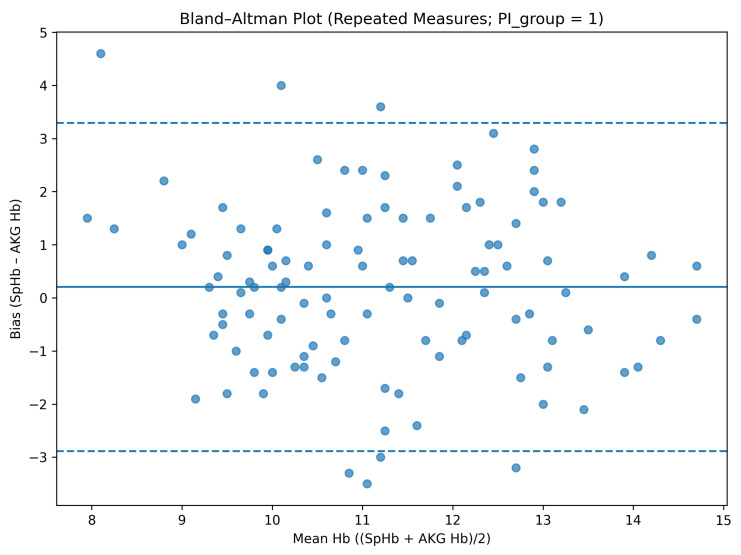
Bland–Altman analysis stratified by perfusion index (PI ≥ 1). The solid line indicates the mean bias, and the dashed lines indicate the 95% limits of agreement (mean bias ± 1.96 SD).

When perfusion index was analyzed as a categorical variable, a borderline association was observed between PI group and bias. Compared with the low-PI group, the high-PI group demonstrated an approximately 0.5 g/dL lower bias (β = −0.49, *p* = 0.069), whereas time point remained non-significant. Inclusion of a PI-by-time interaction term did not improve model fit, and the interaction was not statistically significant (*p* = 0.705); therefore, the main-effects model was retained as the final model (Table 2 and Figure 2, Figure 3 and Figure 4).

### 4.4. Proportional Bias Analysis

Mixed-effects proportional bias analysis showed no significant association between mean hemoglobin level and the SpHb–arterial hemoglobin difference (β = 0.054, *p* = 0.51), indicating the absence of a meaningful proportional bias across the hemoglobin range.

### 4.5. Clinical Error Thresholds and Percent Error

When clinically relevant absolute error thresholds were applied, a substantial proportion of SpHb measurements exceeded ±1.0 g/dL, indicating clinically unacceptable disagreement with arterial hemoglobin values. Errors exceeding ±0.5 g/dL were frequent, while deviations beyond ±1.5 g/dL occurred less commonly but remained clinically relevant. These findings highlight the limited reliability of SpHb measurements for absolute hemoglobin-based decision-making.

Percent error analysis further demonstrated considerable dispersion of SpHb values relative to arterial hemoglobin measurements. Both mean percent error and the median percent error with interquartile range confirmed substantial normalized measurement variability, extending beyond that captured by mean bias and limits of agreement alone.

**Figure 5:**
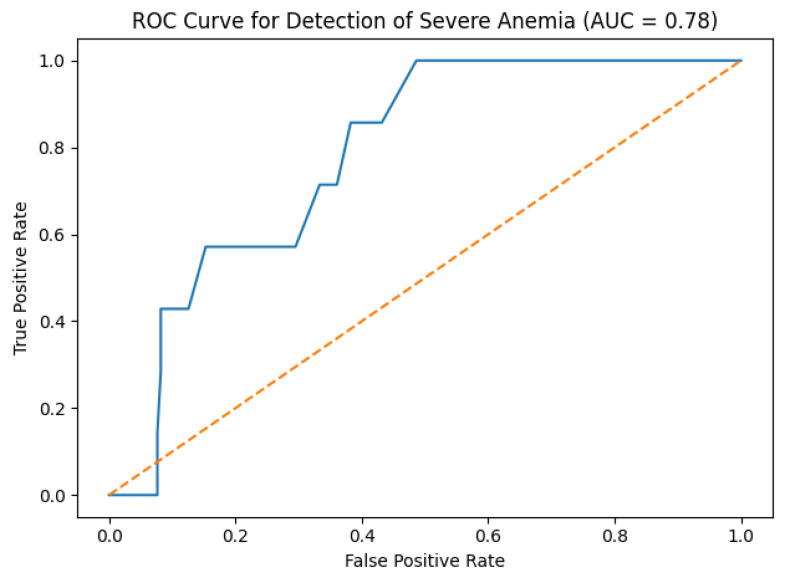
Receiver operating characteristic (ROC) curve for detection of severe anemia. Confidence intervals were derived using patient-level cluster bootstrapping to account for repeated measurements within individuals. Given the low number of severe anemia events, ROC estimates should be interpreted as exploratory. The dotted yellow line indicates the no-discrimination reference line (AUC = 0.50).

Severe anemia, defined as an arterial hemoglobin concentration < 8 g/dL, occurred in 7 of 190 paired measurements. Given the repeated-measures structure and the limited number of severe anemia events, ROC analyses were performed with explicit consideration of uncertainty and within-patient correlation. Using patient-level cluster bootstrapping to derive robust confidence intervals, SpHb demonstrated a moderate discriminative ability for detecting severe anemia, with an area under the curve (AUC) of 0.78 (95% confidence interval [CI], 0.61–0.93). However, given the small number of severe anemia events, these estimates should be interpreted as exploratory and hypothesis-generating rather than definitive measures of diagnostic performance. As a sensitivity analysis to reduce dependence on repeated observations, we repeated the ROC analysis using one clinically relevant observation per patient, defined as the lowest arterial hemoglobin value recorded for each individual (Figure 5).

In this analysis, severe anemia was present in 5 of 60 patients, and the corresponding AUC was 0.71 (bootstrap 95% CI, 0.46–0.89), indicating a directionally similar but less precise discriminative performance. Paired measurements were distributed across the perioperative period, including post-induction, intraoperative, and pre-extubation timepoints.

To provide clinically interpretable performance measures, the positive predictive values (PPVs) and negative predictive values (NPVs) were calculated at clinically relevant SpHb decision thresholds. Given the low prevalence of severe anemia in the study population, PPVs were modest, whereas NPVs were high. Bootstrap-based confidence intervals were calculated for PPV and NPV to reflect the substantial uncertainty associated with the low event rate. These predictive values are prevalence-dependent and should therefore be interpreted cautiously. Accordingly, PPV/NPV estimates are reported for descriptive purposes only and should be considered exploratory.

### 4.6. Trending Ability

**Figure 6:**
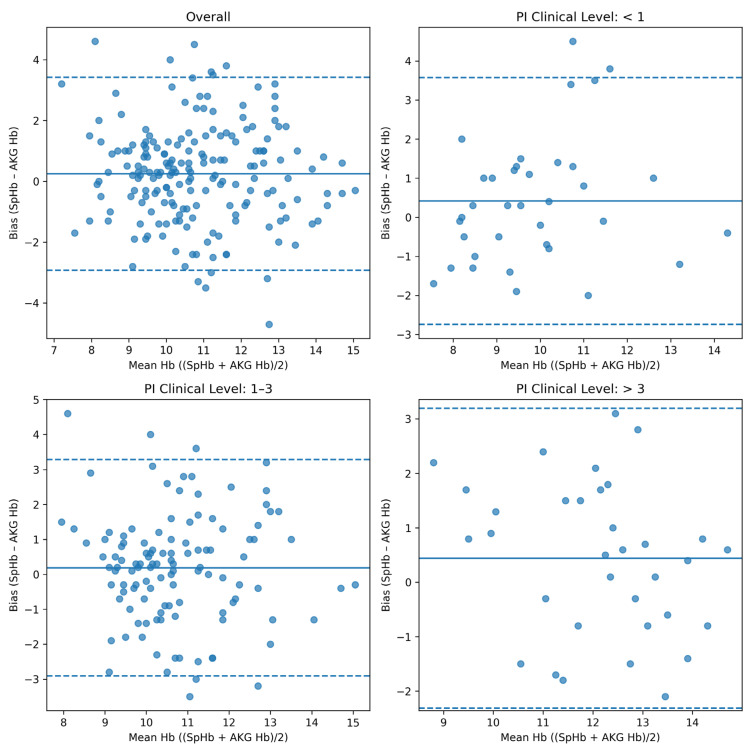
Four-quadrant plot analysis of SpHb trending ability. The solid line indicates the mean bias, and the dashed lines indicate the 95% limits of agreement (mean bias ± 1.96 SD).

Trending performance was evaluated using four-quadrant analysis with an exclusion zone of ±0.5 g/dL. Overall concordance between directional changes in SpHb and arterial hemoglobin was 85.5%, indicating a strong ability of SpHb to track hemoglobin trends over time (Figure 6).

**Figure 7:**
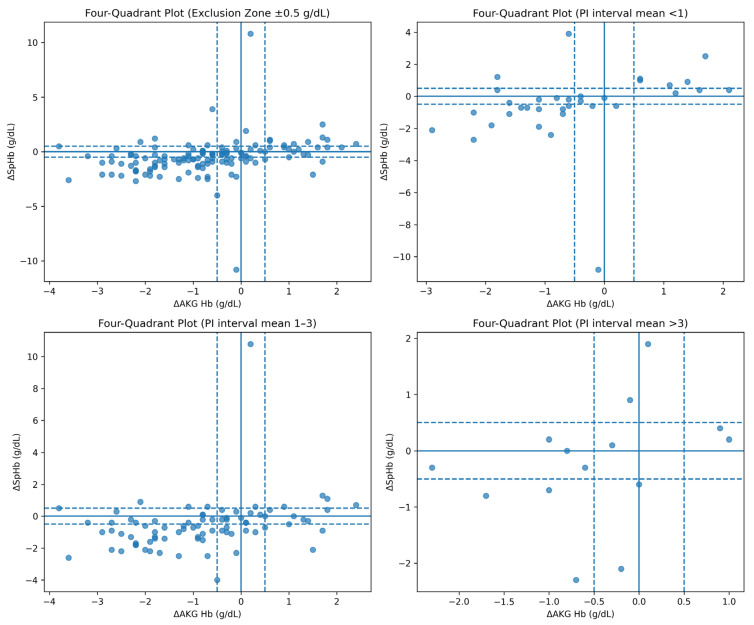
Four-quadrant plot analysis of SpHb trending performance across perfusion index levels. The solid line indicates the mean bias, and the dashed lines indicate the 95% limits of agreement (mean bias ± 1.96 SD).

When stratified by perfusion index, concordance was 86.8% in the low-PI group (37 paired measurements) and 83.9% in the high-PI group (152 paired measurements). Despite reduced absolute agreement under low perfusion conditions, the ability of SpHb to follow directional hemoglobin changes remained preserved across perfusion states (Figure 7).

When clinically relevant absolute error thresholds were applied, 48.4% of SpHb measurements exceeded ±1.0 g/dL and 30.0% exceeded ±1.5 g/dL overall, indicating limited reliability for individual-level hemoglobin-based decision-making. Errors exceeding ±0.5 g/dL were common, while deviations greater than ±1.5 g/dL occurred less frequently but remained clinically relevant. Stratified analyses showed similarly high error rates across perfusion index strata, with higher proportions observed under conditions of reduced peripheral perfusion and in patients receiving vasopressor therapy, underscoring the influence of peripheral perfusion and hemodynamic support on noninvasive hemoglobin measurement accuracy (Table 4).

Notably, the error-threshold analysis (Table 4) showed slightly higher proportions of measurements exceeding ±0.5 and ±1.0 g/dL in the PI ≥ 1 stratum compared with the PI < 1 stratum. This may appear counterintuitive given that PI-stratified Bland–Altman analysis demonstrated wider limits of agreement under low-PI conditions. However, these approaches summarize different aspects of performance: limits of agreement reflect overall dispersion (including tail behavior), whereas threshold exceedance rates are sensitive to the distribution shape around predefined cut-offs and to sampling structure. The PI < 1 subgroup contained only 37 paired measurements, making threshold proportions unstable and highly sensitive to within-patient clustering and repeated-measures imbalance. In addition, PI strata may differ in clinical context (e.g., hemodilution, fluid loading, vasopressor exposure, and hemoglobin range), which could influence absolute error independently of PI. Therefore, PI-related findings should be interpreted as exploratory, with emphasis on the consistent direction of wider agreement limits under low perfusion rather than small differences in threshold proportions.

### 4.7. Clinical Unacceptability of Measurement Error

In mixed-effects logistic regression analysis evaluating clinically unacceptable disagreement (|SpHb − arterial hemoglobin| > 1 g/dL), a low perfusion index was independently associated with a higher probability of clinically relevant measurement error. Time point was not significantly associated with this outcome, indicating that measurement reliability was not systematically affected by procedural duration but was sensitive to perfusion status.

### 4.8. Sensitivity Analysis Restricted to Pre-Specified Sampling Points

To evaluate the robustness of the main findings and to mitigate potential verification and selection bias related to clinically triggered arterial blood gas sampling, a sensitivity analysis was performed including only paired measurements obtained at pre-specified timepoints (baseline before surgical incision and end of procedure). This restricted dataset included 120 paired measurements from 60 patients.

In this sensitivity analysis, SpHb continued to demonstrate a small mean bias relative to arterial hemoglobin, accompanied by wide limits of agreement, consistent with the primary analysis. The proportion of measurements exceeding clinically relevant absolute error thresholds (±1.0 g/dL and ±1.5 g/dL) remained high, indicating limited reliability for absolute hemoglobin determination.

Importantly, the overall direction and magnitude of agreement metrics and error distributions were concordant with those observed in the full dataset analysis. These findings suggest that the observed limitations in absolute accuracy are not solely driven by clinically triggered “high-risk” sampling periods but are also present at standardized perioperative timepoints (Table 5).

### 4.9. Perfusion Index-Stratified Analyses

Although only a small number of patients exhibited low perfusion index values at baseline, repeated intraoperative measurements resulted in a larger number of paired observations under low-PI conditions. Specifically, 22 patients contributed 37 paired measurements to the PI < 1 stratum, whereas 59 patients contributed 152 measurements to the PI ≥ 1 stratum (Table 6). The median number of measurements per patient was comparable across PI strata, indicating that the higher number of low-PI observations was driven by repeated measurements within a subset of patients rather than by a single outlier.

## 5. Discussion

In this study, continuous noninvasive hemoglobin monitoring (SpHb) during the intraoperative period in neurosurgical patients was shown to have limited accuracy for absolute hemoglobin determination when compared with arterial blood gas hemoglobin values; however, it demonstrated clinically meaningful performance in tracking directional changes (trending) in hemoglobin levels. Although the mean bias was small, the wide limits of agreement suggest that SpHb measurements alone are insufficient for decision-making based on absolute hemoglobin values, as previously reported in surgical cohorts demonstrating clinically relevant dispersion and wide limits of agreement [4,6,8,11,16]. Similar discrepancies between absolute agreement and trending performance have also been described in other surgical populations, including maxillofacial surgery, further supporting the cautious interpretation of SpHb for absolute hemoglobin-based decisions [11]. Despite a small mean bias, the wide limits of agreement and the high proportion of measurements exceeding clinically relevant error thresholds indicate that SpHb has limited suitability for absolute hemoglobin-based decision-making at the individual level, particularly in patients with low perfusion or requiring vasoactive support. These findings highlight the importance of error-threshold-based analyses as a complement to agreement metrics when assessing real-world clinical applicability.

The clinical application of SpHb technology has been primarily investigated to support transfusion decision-making in major surgical procedures and in patients at high risk of significant bleeding. The ability to provide continuous and noninvasive measurements offers a time advantage over conventional laboratory-based methods and may increase clinician awareness of ongoing hemoglobin changes [7,12,14]. Nevertheless, the variability in accuracy reported across different clinical scenarios indicates that the role of SpHb in routine clinical practice should be interpreted with caution [9,13,16]. The exploratory subgroup analysis according to anesthetic technique suggested potential differences in the association between SpHb and arterial hemoglobin; however, these observations must be interpreted with substantial caution. The propofol-based TIVA group comprised a very small proportion of the study population, resulting in pronounced group imbalance and limited statistical power.

Consequently, these findings should be considered hypothesis-generating only and do not support definitive conclusions regarding anesthetic-specific effects on SpHb performance. Future studies with balanced group sizes and prospective stratification by anesthetic technique are required to adequately evaluate potential interactions between anesthetic regimen and noninvasive hemoglobin monitoring accuracy An exploratory observation suggested a numerically higher SpHb–arterial hemoglobin correlation in the propofol-based TIVA subgroup compared with volatile anesthesia. However, only five patients received TIVA, resulting in marked group imbalance. Therefore, this subgroup is severely underpowered to detect clinically meaningful differences, and the observed correlation coefficients may be driven by individual patient-level characteristics and selection effects rather than anesthetic technique itself. For this reason, anesthetic-related findings are reported descriptively only and should be interpreted strictly as hypothesis-generating [11].

Previous studies have demonstrated that even clinically relevant discrepancies may exist between blood gas analyzers and central laboratory measurements, indicating that absolute agreement is not consistently achieved even among reference methods for hemoglobin determination [5,18,19,20,21]. Within this context, the lack of exact concordance between SpHb and arterial hemoglobin values may be considered a predictable limitation inherent to noninvasive monitoring technologies [13,16].

The influence of peripheral perfusion on the accuracy of SpHb measurements has been strongly emphasized in the literature. Although perfusion index was associated with increased measurement variability, PI-specific regression findings should be interpreted cautiously. Low PI values were observed in a relatively small proportion of measurements, limiting the stability and precision of stratified estimates. Consequently, while the direction of effect was consistent across analyses, confidence intervals were wide, and PI-related findings should be viewed as exploratory rather than confirmatory. The use of mixed-effects modeling for agreement analysis allowed appropriate handling of repeated measurements and provided more reliable estimates of uncertainty compared with conventional Bland–Altman methods. Reporting confidence intervals for agreement metrics, ROC performance, and trending concordance improves the interpretability and transparency of the findings. Four-quadrant plot analysis was selected to evaluate trending ability because it focuses on the clinically relevant question of whether a monitoring device correctly captures the direction of hemoglobin change over time, rather than absolute agreement. The predefined exclusion zone further ensured that concordance estimates were not driven by trivial fluctuations without clinical relevance.

Under conditions of impaired perfusion—particularly in the presence of vasoconstriction, hypothermia, or hemodilution—the reliability of noninvasive hemoglobin measurements has been shown to decrease [11,16]. Consistent with these findings, our study demonstrated wider limits of agreement and a higher rate of clinically unacceptable measurement error in patients with a low perfusion index [11,13,16].

High crystalloid infusion rates and volume loading during surgery may further affect peripheral tissue perfusion and the optical principles underlying SpHb measurement, thereby contributing to measurement discrepancies. Previous studies have shown that different infusion fluids can influence the accuracy of SpHb readings, with rapid volume replacement in particular being associated with reduced measurement reliability [11,15]. The patient population in our study, which was exposed to substantial intraoperative volume administration, supports the clinical relevance of this interaction.

The relatively high prevalence of preoperative anemia observed in this cohort should be interpreted in the context of perioperative hemoglobin status rather than as evidence of chronic anemia. Patients with documented chronic anemia were excluded by design based on predefined operational criteria. Therefore, baseline anemia in the present study reflects transient or perioperative hemoglobin values commonly encountered in neurosurgical populations. Anemia and fluctuations in hemoglobin levels are critical determinants of cerebral oxygen delivery, particularly in neurosurgical patients. It is well established that anemia may impair cerebral oxygen metabolism and increase the risk of secondary brain injury [3]. Therefore, early detection of abrupt intraoperative hemoglobin changes may be clinically valuable, even in the presence of limited absolute measurement accuracy [7,12].

Receiver operating characteristic analysis suggested that continuous noninvasive hemoglobin monitoring provides a moderate ability to discriminate severe anemia during neurosurgery. However, severe anemia events were relatively infrequent in our cohort, resulting in limited statistical power and wide confidence intervals around ROC-based estimates. Accordingly, these findings should be interpreted strictly as exploratory and hypothesis-generating.

Although patient-level cluster bootstrapping and sensitivity analyses restricted to one observation per patient improved robustness, they do not eliminate the fundamental limitations imposed by the low event rate. Moreover, SpHb measurements are known to be influenced by peripheral perfusion, hemodilution, and intraoperative physiological variability, all of which may further affect reliability for absolute hemoglobin classification.

Therefore, while ROC findings indicate that SpHb has some capacity to discriminate severe anemia, these results do not support its use as a standalone diagnostic test. Rather, SpHb should be regarded as an adjunctive monitoring modality that complements invasive or laboratory-based hemoglobin measurements, particularly by providing continuous real-time information on hemoglobin trends during surgery [9,13,16,17].

In contrast, the high concordance rates observed in four-quadrant analysis demonstrate that SpHb provides reliable information regarding hemoglobin trends over time. Prior studies conducted in surgical populations and perioperative cohorts have similarly shown that SpHb can accurately reflect directional changes in hemoglobin levels despite limitations in absolute accuracy [12,14,16,22]. This characteristic renders SpHb particularly valuable as an early warning tool in surgical procedures associated with a high risk of bleeding [4,7,16].

The cohort demonstrated substantial blood loss and high crystalloid administration rates, suggesting that the study population may represent a higher-risk subset of elective neurosurgery rather than routine low-bleeding elective cranial cases. Such hemodynamic variability, hemodilution, and vasoconstrictive responses may negatively affect SpHb agreement, and therefore the present findings should be generalized cautiously to standard elective procedures with minimal blood loss.

In conclusion, SpHb monitoring showed limited reliability for absolute hemoglobin assessment at the individual level, as reflected by wide limits of agreement and frequent clinically unacceptable errors. Nevertheless, SpHb preserved directional trending ability and may serve as an adjunctive early warning tool during neurosurgery, particularly when interpreted alongside perfusion index and hemodynamic context. SpHb should not be used as a standalone diagnostic method for transfusion or anemia management, and invasive or laboratory confirmation remains essential. Any anesthetic technique-related differences observed in this cohort should be considered exploratory and require confirmation in larger, prospectively stratified studies [9,11,13,16].

An additional consideration is that the baseline hemoglobin measurement was obtained after induction of anesthesia and initial fluid administration rather than as a true pre-induction laboratory value. Therefore, the relatively high prevalence of hemoglobin < 12 g/dL at baseline may partly reflect peri-induction hemodilution rather than chronic anemia. This timing should be considered when interpreting agreement and diagnostic performance analyses across subsequent perioperative measurements.

The relatively high prevalence of preoperative anemia reflects perioperative hemoglobin values and should not be interpreted as chronic anemia, as patients with documented chronic anemia were excluded by design. Although SpHb demonstrated limited agreement with arterial hemoglobin for absolute value determination, it provided useful information for tracking directional hemoglobin changes over time. Accordingly, SpHb should not be considered interchangeable with arterial blood gas hemoglobin measurements for absolute decision-making.

In addition, it should be emphasized that the laboratory hematology analyzer (complete blood count, CBC) is generally considered the reference standard for hemoglobin measurement. Arterial blood gas analyzers, while clinically practical intraoperatively, have their own analytical variability related to calibration, sample handling, and co-oximetry methodology. Therefore, using ABG hemoglobin as the reference may introduce measurement error that can widen the apparent limits of agreement between SpHb and ‘reference’ hemoglobin. Postoperative laboratory CBC hemoglobin values were available for a subset of patients as part of routine care; however, these values were not consistently time-matched to intraoperative paired measurements and were therefore not suitable for formal cross-validation analyses.

Although arterial blood gas analysis is widely used as a reference method for perioperative hemoglobin assessment, it is not entirely free from analytical variability. Hemoglobin measurement by blood gas analyzers is based on co-oximetry principles and may be influenced by calibration procedures, sample handling, hemodilution, and the presence of dyshemoglobins. Previous studies have reported small but clinically relevant discrepancies between blood gas-derived hemoglobin values and central laboratory hematology analyzers [5,18,19,20,21]. Therefore, part of the observed disagreement between SpHb and arterial hemoglobin measurements in the present study may also reflect methodological limitations inherent to the reference technique itself rather than measurement error attributable exclusively to the non-invasive device.

### Limitations

This study has several limitations. First, it was conducted at a single center with a relatively limited sample size, which may restrict the generalizability of the findings. Second, because the study population consisted exclusively of patients undergoing elective neurosurgical procedures, the results cannot be directly extrapolated to other types of surgery, emergency interventions, or critically ill patients in intensive care settings.

Third, because SpHb measurements are dependent on peripheral perfusion, measurement accuracy may have been affected, particularly in patients with low perfusion index values. High intraoperative crystalloid administration and hemodilution may have further contributed to variability in noninvasive hemoglobin measurements. In addition, only a single noninvasive hemoglobin monitoring system was evaluated in this study; therefore, the findings may not be generalizable to other devices or technologies.

Finally, arterial blood gas hemoglobin measurements were used as the reference standard; however, this method itself may be subject to measurement variability when compared with central laboratory-based assays. Consequently, some of the observed discrepancies may be attributable to technical limitations inherent to the measurement methods.

An additional limitation relates to perfusion index-stratified analyses. When PI dropped below 1, sensor repositioning and/or device recalibration was performed in accordance with manufacturer recommendations. Consequently, measurements classified under low-PI conditions may include post-intervention values rather than untreated low-perfusion states. This limits interpretation of intrinsic device performance under sustained low-perfusion conditions, and PI subgroup findings should therefore be regarded as exploratory.

An additional limitation relates to the indication-based arterial hemoglobin sampling strategy. Because arterial measurements were obtained when clinically indicated, sampling was not strictly random and may have preferentially occurred during periods of hemodynamic instability or suspected bleeding. This may introduce verification and selection bias, potentially inflating measurement disagreement and influencing ROC-based diagnostic estimates. However, this approach reflects routine intraoperative clinical practice and allows evaluation of SpHb performance under conditions where accurate hemoglobin assessment is most clinically relevant. Consequently, absolute agreement and diagnostic metrics should be interpreted with caution, whereas trend monitoring findings may be more robust in this context.

## 6. Conclusions

In conclusion, continuous noninvasive hemoglobin monitoring showed limited reliability for absolute hemoglobin assessment at the individual level, as reflected by wide measurement dispersion. However, its ability to track hemoglobin trends may offer clinically useful adjunctive information during neurosurgical procedures. The interpretation of SpHb values should be cautious, particularly in the presence of impaired peripheral perfusion or hemodynamic instability, and critical decisions should continue to rely on invasive or laboratory-based hemoglobin measurements.

## Figures and Tables

**Table 1:** Baseline demographic, clinical, surgical, hemodynamic, and hemoglobin characteristics of the study population (n = 60).

Parameter	Value
Demographic Characteristics	
Age, years	60.8 ± 13.9
Female sex, n (%)	30 (50.0)
Weight, kg	77.8 ± 12.9
Height, cm	166.1 ± 9.2
Estimated total blood volume, mL	4587 ± 550
ASA physical status I–II, n (%)	39 (65.0)
ASA physical status III, n (%)	21 (35.0)
Number of comorbidities	1.55 ± 1.22
Surgical And Anesthetic Characteristics	
Duration of surgery, min	92.6 ± 28.4
Preoperative fasting time, h	10.4 ± 2.3
Volatile anesthesia, n (%)	55 (91.7)
Total intravenous anesthesia (TIVA), n (%)	5 (8.3)
Hemodynamic And Volume-Related Parameters	
Estimated blood loss, mL	1000.0 ± 707.5
Blood loss ≥20% of total blood volume, n (%)	26 (43.3)
Total crystalloid administration, mL	2836.7 ± 1094.9
Crystalloid rate, mL/kg/h	30.8 ± 13.3
Crystalloid rate >10 mL/kg/h, n (%)	59 (98.3)
Vasopressor use, n (%)	8 (13.3)
Hemoglobin And Perfusion-Related Variables	
Preoperative arterial hemoglobin, g/dL	11.7 ± 1.8
Preoperative anemia (hb < 12 g/dL), n (%)	31 (51.7)
Mean perfusion index (PI)	2.83 ± 1.55
Low perfusion index at baseline (PI < 1), n (%)	3 (5.0)

Data are presented as mean ± standard deviation or number (percentage), as appropriate.

**Table 2:** Mixed-effects model and repeated-measures Bland–Altman results.

Analysis	Effect/Metric	Estimate	95% CI/LoA	*p*-Value
**Mixed-effects model (A1)**	PI group (High vs. Low)	β = −0.49	−1.02 to 0.04	0.069
**Mixed-effects model (A1)**	Time point	—	—	0.873
**Random effect**	Between-patient variance	—	—	≈0.001
**Interaction model (A2)**	PI × Time interaction	β = 0.069	—	0.705
**Bland–Altman (overall)**	Mean bias	+0.23 g/dL	—	—
**Bland–Altman (overall)**	Limits of Agreement	—	−3.26 to +3.72	—
**Study size**	Repeated measures	190 measurements	60 patients	—

β denotes fixed-effect coefficients from linear mixed-effects models. LoA indicates limits of agreement derived from repeated-measures Bland–Altman analysis. The primary model was A1, as the interaction model (A2) did not improve model fit.

**Table 3:** Bland–Altman agreement stratified by perfusion index.

PI Group	Mean Bias (g/dL)	Total Sd (g/dL)	Lower LoA (g/dL)	Upper LoA (g/dL)
**Low PI**	0.32	1.97	−3.54	4.18
**High PI**	0.16	1.65	−3.07	3.39

LoA indicates limits of agreement derived from repeated-measures Bland–Altman analysis.

**Table 4:** Proportion of sphb measurements exceeding clinically relevant absolute error thresholds.

Subgroup	n (Measurements)	>±0.5 g/dL	>±1.0 g/dL	>±1.5 g/dL
**Overall**	190	72.1%	48.4%	30.0%
**Perfusion Index (PI)**				
**PI < 1 (low PI)**	37	65.8%	45.6%	27.8%
**PI ≥ 1 (higher PI)**	152	76.6%	50.5%	31.5%
**Vasopressor use**				
**No**	121	74.4%	47.1%	28.1%
**Yes**	69	68.1%	50.7%	33.3%

Absolute error was defined as |SpHb − arterial hemoglobin|. Clinically meaningful thresholds (±0.5, ±1.0, and ±1.5 g/dL) were prespecified based on commonly used perioperative validation criteria.

**Table 5:** Distribution of paired SpHb–ABG measurements across perioperative phases.

Perioperative Phase	Number of Paired Measurements, n (%)
Pre-incision (baseline)	60 (31.6%)
Intraoperative (clinically indicated)	70 (36.8%)
End of procedure (pre-extubation)	60 (31.6%)
**Total**	**190 (100%)**

Paired measurements were classified according to the timing of arterial blood gas sampling. Baseline samples were obtained before surgical incision, end-of-procedure samples immediately prior to extubation, and intraoperative samples were obtained when clinically indicated (e.g., suspected bleeding, hemodynamic instability, or at the discretion of the attending anesthesiologist).

**Table 6:** Patient-level contribution to perfusion index strata.

PI Stratum	n Patients	n Measurements	Measurements Per Patient (Min–Median–Max)
PI < 1	22	37	1–2–3
PI ≥ 1	59	152	1–2–6

The table summarizes the number of unique patients contributing measurements to each perfusion index (PI) stratum and the distribution of repeated measurements per patient. Although low PI values (PI < 1) were observed in a limited number of patients at baseline, multiple measurements per patient occurred during the intraoperative period, accounting for the higher number of paired observations in the low-PI stratum.

## Data Availability

The original contributions presented in this study are included in the article. Further inquiries can be directed to the corresponding author.

## Abbreviations

ABG	Arterial Blood Gas
ASA	American Society of Anesthesiologists
AUC	Area Under the Curve
BE	Base Excess
BMI	Body Mass Index
CO	Carbon Monoxide
EtCO_2_	End-Tidal Carbon Dioxide
FiO_2_	Fraction of Inspired Oxygen
Hb	Hemoglobin
ICP	Intracranial Pressure
LoA	Limits of Agreement
MAC	Minimum Alveolar Concentration
MAP	Mean Arterial Pressure
PCO_2_	Partial Pressure of Carbon Dioxide
PEEP	Positive End-Expiratory Pressure
PI	Perfusion Index
PO_2_	Partial Pressure of Oxygen
ROC	Receiver Operating Characteristic
SD	Standard Deviation
SpHb	Continuous Noninvasive Hemoglobin
SpO_2_	Peripheral Oxygen Saturation
TIVA	Total Intravenous Anesthesia
